# The benefits of integrated systems for managing *both *samples *and *experimental data: An opportunity for labs in universities and government research institutions to lead the way

**DOI:** 10.1186/1759-4499-3-2

**Published:** 2011-06-27

**Authors:** Rory Macneil

**Affiliations:** 1 Axiope Limited, 24 Fountainhall Road, Edinburgh EH9 2LW, UK

## Abstract

Currently most biomedical labs in universities and government funded research institutions use paper lab notebooks for recording experimental data and spreadsheets for managing sample data. One consequence is that sample management and documenting experiments are viewed as separate and distinct activities, notwithstanding that samples and aliquots are an integral part of a majority of the experiments carried out by these labs.

Various drivers are pushing labs towards integrated management of sample data and experimental data. These include the ever increasing amounts of both kinds of data, the increasing adoption of online collaborative tools, changing expectations about online communication, and the increasing affordability of electronic lab notebooks and sample management software. There is now an opportunity for smaller labs, which have been slow to move from paper to electronic record keeping, to leapfrog better resourced commercial labs and lead the way in adopting the new generation of tools which permit integrated management of samples and experimental data and a range of tangible benefits to conducting research, including:

1. Fewer lost and mislabelled samples

2. Clearer visualization of relationships between samples and experiments

3. Reduction of experimental error

4. More effective search

5. Productivity gains

6. More efficient use of freezers, leading to cost reduction and enhanced sustainability

7. Improved archiving and enhanced memory at the lab and institutional levels

## Introduction

Traditionally three kinds of software have been used by commercial labs to manage physical things, like samples, and abstract things, i.e. information in electronic form like spreadsheets and thumbnails of images. The first kind of software, *Electronic lab notebooks *(ELNs,) has been used to manage information.

Terminological confusion, however, besets discussion about the other two kinds of software [1]. The term *Laboratory Information Management Systems *(LIMS) is sometimes used in a broad sense to mean "Software applications used to automate the routine operations of a laboratory" [2], or instrument data management. It is also used, however, to specifically include sample management. Thus the Wikipedia entry on LIMS starts by stating, "The core function of LIMS is the management of samples" [3]. The confusion arises because although some LIMS do indeed deal with sample management in addition to assisting with the automation of other functions like QA, QC, and workflow, there is a third category of software, usually referred to as *sample or inventory management software*, that deals *only *with managing samples. Unlike ELN or LIMS, there is no generally accepted acronym to describe this third kind of software. Sample/inventory management software is often included in lists of LIMS vendors, with no distinction made between them and the fully fledged LIMS which handle a full range of lab automation functions, including various forms of instrument data management.

Recently there has been considerable discussion about the coming together of LIMS and ELNs. This discussion often glosses over the terminological confusion noted above. When you look beneath the surface, what is being pointed to is the emergence of systems that are able to handle both general data management and sample management [4]. I.e. what is converging with ELNs is really the sample/inventory management function, handled by LIMS and sample/inventory management software with ELNs, rather than the other aspect of LIMS, instrument data management.

This article looks at the needs driving the push for integration of sample management and management of experimental data, and the developments in software that are beginning to make this integration possible. It discusses the different practices, needs and usage of software in commercial labs, where usage of ELNs and fully fledged LIMS is pervasive, and labs in academic settings and small-to-medium size biotechs, which are just beginning to look at ELNs and LIMS. The article concludes that these smaller labs should find it easier to adopt the new integrated solutions which are becoming available because these labs do not face the complication of attempting to make the new solution work together with a legacy ELN and/or LIMS. There is thus an opportunity for labs in universities and government research institutions, which have been slow to move from paper to electronic record keeping, to leapfrog better resourced commercial labs and lead the way in adopting the new generation of tools which permit integrated management of samples and experimental data.

## The current state of sample and experimental data management in a typical academic lab

### Sample management

The majority of labs in the fields of biology, chemistry and medicine deal with samples in their research. These samples are of many kinds, e.g. blood, DNA, bacteria, etc., and fall into various categories, e.g. clinical and non-clinical. A lot is done to the samples, e.g. they are split into aliquots, shipped between facilities, checked in and out of freezers, counted, analyzed, and used in experiments.

Managing samples is a core function for these labs, and it is not be possible for the researchers who work in the labs to realize their core mission-carrying out experiments-without effective sample management. The physical side of sample management is highly standardized: samples are most often place in trays of standard configurations inside boxes, also of standard configurations, inside freezers, also of standard configurations. In many academic labs, keeping track of where a sample is, its history, whether it has been aliquoted, and other information about the sample is done by hand on a paper record, typically placed near the freezer and/or recorded in spreadsheets.

Depending on the size of the lab, the nature of the research, and where the lab falls on the spectrum of group-oriented to individual researcher-oriented, sample management may be handled by one or two people in the lab, or it may be something that many or most people do themselves from time to time. Thus one person or, more often, multiple people have access to the sample record and participate in its development.

In this environment, basic sample management needs include:

• To store all sample information, aliquot numbers, dates, web links and images

• To set alerts

• To generate reports

• To graphically display containers containing samples

• To name containers

• To assign roles-who can do what with which categories of samples

### Experimental data management

For labs that use samples as an integral part of their research, managing samples is a vital task, but it is only a means to an end, conducting experiments. Samples, and analysis of samples, are used in experiments. Experiments are often carried out on the basis of protocols. They are usually documented with a mix of paper lab notebooks and things in electronic form like spreadsheets, Word documents, PDFs, and images in scientific formats. In addition to information specifically relating to experiments, labs also record general information like meeting notes. This general information, but not the data relating specifically to experiments, is increasingly created and shared within an online collaborative tool like a wiki.

With sample management a few well defined roles usually provides sufficient differentiation to reflect the differentiation of labor in the lab. When it comes to general information sharing and in particular management of experimental data, however, a finer grained controls system is needed, for example so that each individual in the lab can have their own completely private space, some records can be shared by specified groups or between the PI and a student, and some records can be seen by everyone in the lab.

### Barriers to adoption of integrated management of samples and experimental data

The above picture of how labs in universities and government research institutions deal with experimental data and sample data reinforces the point that they have been slow to adopt electronic lab notebooks and sample management software. There are a variety of reasons for this, prominent among them inertia and the difficulty of making decisions in a consensual environment [5]. Other factors include price, the simplicity, flexibility, convenience and familiarity of paper notebooks and spreadsheets, conservatism on the part of PIs, and concerns about being tied in to proprietary file formats which might make data inaccessible in the future and/or how to ensure that records kept electronically enable compliance with regulations like 21 CFR part 11 [6].

## Drivers behind the push to integrate management of samples and experimental data

Notwithstanding these barriers, a number of factors are driving the need to integrate the two core activities of the lab -- managing samples and conducting and documenting experiments.

An important background factor is the *ever increasing amount of data to process and ever mounting pressure to process it faster*. This puts a premium on more efficient management of information generally. It is bad enough if your lab does not have efficient mechanisms for managing sample data and/or managing experimental data at a time when the number and complexity of both kinds of data is continually expanding. But given that samples are relevant to most of the experiments being carried out, it is even worse if you manage these two kinds of data separately.

A second factor is the *increasing amount of time spent by researchers online as a percentage of total time spent at the computer*. This is true in terms of time spent working-a recent study of seven life sciences labs noted that most now use wikis to share general information like meeting notes and non-confidential things like protocols, and that the scientists in the labs were big users of Google, for search, but also of Google Docs, and of online databases [7]. The growing popularity of Mendeley for reference management is an example of adoption of a specialist online tool for researchers. Researchers also are spending more time online outside of work, where scientists are just as likely as others to use Google for search, Facebook for communicating with friends and family, etc.

The changing landscape of tools that researchers use at work and outside work, and the changing pattern of how people are using these tools, is giving rise to a third factor, *changing expectations about how information should be discovered and managed*, and the kinds of tools that are appropriate-and necessary-to do this efficiently. Expectations are not only changing, they are being raised. When you are used to Google and Facebook it seems odd not be able to, say, link information about a particular sample used in an experiment to the write up of that experiment. This trend is accelerating as a new generation of postdocs, graduate students and undergraduates, who are comfortable with online tools and take for granted their ongoing rapid development, take active roles in labs.

A fourth factor, mirroring the increase in data and its growing, complexity, is *the decrease in the cost of accessing tools to manage the data*. 10 years ago, LIMS and ELNs cost $10,000s-$100,000s and were beyond the reach of virtually all academic labs. Five years ago, low cost ELNs and sample/inventory management software began appearing on the market. Today free-admittedly simple-ELNs and sample/inventory management software, is becoming available, and fully featured ELNs and sample/inventory management systems are available for as little as $1,000. LIMS with instrument automation capability are the exception to this trend, and remain prohibitively expensive for virtually all academic labs.

A fifth factor arises from *the role samples play and the way in which they are used in experimental research*. Take an antibody, for example. It might begin life as a sample the lab brings in or creates. Its original characteristics are recorded. In many cases it will be aliquoted. Then the aliquots are processed or analyzed, and the changes they undergo are also recorded, and analyzed. The aliquots and what has happened to them may be compared with others and what happened to them, and this process will be examined in the broader context of what else was going on in the experiment. Does the management of data relating to the sample fall under the category of sample management or experimental data management? Both, of course! The distinction between the two is entirely artificial, and only arose because of the lack of tools that allowed the sample/aliquots and their history to be viewed in the context of the experiment(s) in which they were used.

## What does integrated management of samples and experimental data involve in practice?

Integration of sample management and management of experimental data could take a variety of forms. To deliver the benefits of this integration described in the following section, a tool or system needs to have the following characteristics and capabilities:

1. A structure or framework capable of dealing with both (a) samples and (b) experimental data and other information relevant to the lab's research such as protocols and meeting notes.

2. A unified interface that presents sample data and experimental data/other information in an intuitive and user-friendly manner.

3. The ability to associate sample data with experimental data/other information.

4. The ability to search for all information including sample data and experimental data, just sample data, and just experimental data.

5. A fully fledged sample management system which meets the basic sample management needs, i.e.

• Storage of all sample information, aliquot numbers, dates, web links and images

• Setting alerts

• Generating reports

• Graphic display of containers with samples

• Naming containers

• Assigning roles-who can do what with which categories of samples

6. A fully featured electronic lab notebook, which supports:

• Creating and importing research data

• Putting structure into research data

• Controlled sharing of data between individuals and groups

• A messaging system

## Benefits of integrated management of samples and experimental data

Integrated management of samples and experimental data will bring important benefits to academic labs. These include:

### Fewer lost and mislabelled samples

Use of sample/inventory management software results in better sample management, including fewer lost and mislabelled samples. Beyond this, *integrating management of samples and experimental data also leads to better quality sample management*. This is because the ability to easily associate sample data with other data, e.g. the record of an experiment in which the sample has been used, adds a second context in which the sample can be identified. This acts as a kind of information quality control mechanism or checking system. If a sample is missing or misidentified, in addition to looking for it in the sample management system, the experimental record can be searched.

### Clearer visualization of relationships between samples and experiments

Since sample data and experimental data are recorded in one integrated system, it is possible to make one or multiple links between the record showing where the sample is stored, and its history, and the experiments in which it has been used.

### Reduction of experimental error

The ability to link sample data to experimental data, resulting in few lost and misplaced samples and an improved ability to visualize experiments, leads in turn to fewer mistakes being made when experiments are conducted, and to errors that are recorded being discovered earlier in the experimental process, so that they can be corrected or procedures rerun, and the revised results can be used in drawing conclusions and in publication of results.

### More effective search

Since sample data and experimental data are all in the same system, it's possible to conduct unified searches, e.g. you could search for all records which contain sample 'x' in all ELISA experiments. This is a big step forward from having to separately search in the sample/inventory management system for a sample or set of samples, searching in the ELN for ELISA experiments, and then trying to match the two together manually.

### Higher quality analysis

Reduction of experimental error and more effective search lead in turn to higher quality analysis.

### Productivity gains

Productivity gains from using an integrated system should be substantial:

• Regardless of whether sample management is handled by one person for the whole lab, or multiple people are involved, the system will be more orderly and better understood, leading to more effective sample management.

• Higher quality search will lead to experiments being carried out more quickly, and less time being wasted on looking for samples and other data.

• Higher quality analysis will lead to more successful experiments, and more being learned from experiments that fail, leading in turn to more rapid production of papers and additional time to spend on grant preparation.

### Improved archiving and enhanced memory at the lab and institutional levels

Loss of information when a person leaves the lab has been a chronic problem for many years. Leaving scattered slips of paper in desk drawers about the contents of the freezer is not an adequate means of recording inventory but that's frequently what happens. The next person continuing the project spends a lot of time reconstructing the previous work before work can continue. With an integrated online sample and experimental data management system a permanent, searchable record of what's in freezers is created and available to a lab administrator, and anyone else who is has permission to see the system, of all samples that have been created and used by current and past lab members. This can be extended to groups of labs and institutions if they adopt the same integrated solution.

### Sustainability

More efficient management of samples ties in with, and facilitates, more efficient use and maintenance of freezers. Since lab freezers are heavy users of electricity, their efficient use has important implications for environmental sustainability, as is being demonstrated by initiatives like the Store Smart program at UC Davis [8].

## Conclusion

Labs in universities and government research institutions have been slow to adopt electronic lab notebooks and sample management software. There are a variety of reasons for this, prominent among them inertia and the difficulty of making decisions in a consensual environment, and cost. As noted above, the availability of affordable solutions is rapidly removing cost as a factor.

Precisely because labs in publicly funded environments have not had the resources until now to purchase the expensive commercial ELNs and LIMS that have been widely adopted by the pharmaceutical industry, these labs do not face the complication of attempting to make the new solution work together with a legacy ELN and/or LIMS. Somewhat ironically, this now opens an opportunity for smaller labs, which have been slow to move from paper to electronic record keeping, to leapfrog better resourced commercial labs and lead the way in adopting the new generation of tools which permit integrated management of samples and experimental data, and from that a range of tangible benefits to conducting research.

## Competing interests

The author is a shareholder in Axiope, which provides eCAT, an electronic lab notebook with the capability to manage samples. As such he would benefit indirectly from positive responses to this article.
